# Slaughter-House Examinations

**Published:** 1882-10

**Authors:** 


SLAUGHTER-HOUSE EXAMINATIONS.—In the City of Augsburg there were slaughtered during 1881, 66,731 animals for the market. Of these 12,269 were held, among whom were found 246 beeves and 3 hogs with tuberculosis. This makes a proportion of 2.01 per cent., which is much smaller than is alleged to be the case in American cattle.

The most numerous lesion was that produced by distomata (the *Egelwurmer*) in the liver. These were found in 1,061 beeves and 165 sheep.

We may add here that among 168,579 hogs slaughtered at Berlin in 1881 there were found only 128 with trichinæ. In Hamburg there were 73,113 American swine, hams and ribs examined; of these, 695 or 0.95 per cent., were trichinous. Among 55,799 European hogs and pieces there were only two found trichinous, or 0.004 per cent. We fear, however, that there was some national bias in the above comparative examinations.

